# Automated Classification of Lung Cancer Subtypes Using Deep Learning and CT-Scan Based Radiomic Analysis

**DOI:** 10.3390/bioengineering10060690

**Published:** 2023-06-06

**Authors:** Bryce Dunn, Mariaelena Pierobon, Qi Wei

**Affiliations:** 1 Department of Bioengineering, George Mason University, Fairfax, VA 22030, USA; bdunn6@gmu.edu; 2 School of Systems Biology, Center for Applied Proteomics and Molecular Medicine, George Mason University, Fairfax, VA 22030, USA

**Keywords:** deep learning, radiomics, CT tumor segmentation, lung cancer, classification

## Abstract

Artificial intelligence and emerging data science techniques are being leveraged to interpret medical image scans. Traditional image analysis relies on visual interpretation by a trained radiologist, which is time-consuming and can, to some degree, be subjective. The development of reliable, automated diagnostic tools is a key goal of radiomics, a fast-growing research field which combines medical imaging with personalized medicine. Radiomic studies have demonstrated potential for accurate lung cancer diagnoses and prognostications. The practice of delineating the tumor region of interest, known as segmentation, is a key bottleneck in the development of generalized classification models. In this study, the incremental multiple resolution residual network (iMRRN), a publicly available and trained deep learning segmentation model, was applied to automatically segment CT images collected from 355 lung cancer patients included in the dataset “Lung-PET-CT-Dx”, obtained from The Cancer Imaging Archive (TCIA), an open-access source for radiological images. We report a failure rate of 4.35% when using the iMRRN to segment tumor lesions within plain CT images in the lung cancer CT dataset. Seven classification algorithms were trained on the extracted radiomic features and tested for their ability to classify different lung cancer subtypes. Over-sampling was used to handle unbalanced data. Chi-square tests revealed the higher order texture features to be the most predictive when classifying lung cancers by subtype. The support vector machine showed the highest accuracy, 92.7% (0.97 AUC), when classifying three histological subtypes of lung cancer: adenocarcinoma, small cell carcinoma, and squamous cell carcinoma. The results demonstrate the potential of AI-based computer-aided diagnostic tools to automatically diagnose subtypes of lung cancer by coupling deep learning image segmentation with supervised classification. Our study demonstrated the integrated application of existing AI techniques in the non-invasive and effective diagnosis of lung cancer subtypes, and also shed light on several practical issues concerning the application of AI in biomedicine.

## 1. Introduction

Lung cancer is the leading cause of cancer-related death in the United States [1,2]. Computed tomography (CT) imaging remains one of the standard-of-care diagnostic tools for staging lung cancers. However, the conventional interpretation of radiological images can be, to some degree, affected by radiologists’ training and experience, and is therefore somewhat subjective and mostly qualitative by nature. While radiological images provide key information on the dimensions and extent of a tumor, they are unsuitable for assessing clinical–pathological information (e.g., histological features, levels of differentiation, or molecular characteristics) that is critical for the treatment selection process. Thus, the process of diagnosing cancer patients often requires invasive and sometime risky medical procedures, such as the collection of tissue biopsies. Finding new solutions for collecting critical microscopic and molecular features with non-invasive and operator-independent approaches remains a high priority in oncology. The development of reliable, non-invasive computer-aided diagnostic (CAD) tools may provide novel means to address these problems.

**Figure 1:**
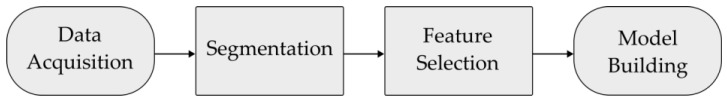
Diagram describing the process of the radiomic machine learning workflow. First, CT image scans are acquired. Tumor regions are delineated during segmentation. Mathematical features are extracted from the tumor segmentations using software tools. These features are used to train machine learning models to make predictions using new data.

Image digitalization coupled with artificial intelligence is emerging as a powerful tool for generating large-scale quantitative data from high-resolution medical images and for identifying patterns that can predict biological processes and pathophysiological changes [3]. Preliminary studies have suggested that objective and quantitative structures that go beyond conventional image examination can predict the histopathological and molecular characteristics of a tumor in a non-invasive way [4]. Several new tools are now available for image analysis, and the machine learning processing pipeline enables automatic segmentation, feature extraction, and model building (Figure 1).

Segmentation is a critical part of the radiomic process, but it is also known to be challenging. Manual segmentation is labor-intensive and can be subject to inter-reader variability [5,6]. To improve segmentation efficiency and accuracy, the development of automated or semi-automated segmentation methods has become an active area of research [7]. Several deep learning models have been used to segment lung tumors from CT scans. However, validation of the reproducibility of these proposed methods using large datasets is still limited [8,9,10,11], and this has hindered their application in clinical settings. Deep learning tools such as U-Net and E-Net have been previously used to automatically segment non-small cell lung tumors and nodules in CT images, but these models were not specifically trained using lung cancer patient data [8].

The incremental multiple resolution residual network (iMRRN) is one of the best performing deep learning methods to have been developed for volumetric lung tumor segmentation from CT images [8,9,10,12]. The iMRRN extends the full resolution residual neural network by combining features at multiple image resolutions and feature levels. It is composed of multiple blocks of sequentially connected residual connection units (RCU), which in turn are convolutional filters used at each network layer. Due to its enhanced capability in recovering input image resolution, the iMRRN has been shown to outperform other neural networks commonly used for segmentation, such as Segnet and Unet, in terms of segmentation accuracy, regardless of tumor size, and localization [7,8,13,14]. Additionally, the iMRRN has also been shown to produce accurate segmentations and three-dimensional tumor volumes when compared with manual tracing by trained radiologists [8]. Its excellent segmentation performance and its public availability make the iMRRN an excellent candidate for other researchers to use.

In this study, we assessed the extent to which the iMRRN coupled with supervised classification can predict lung tumor subtypes based on CT images acquired from lung cancer patients. Segmentation performance was used to inform improvements to the tumor delineation process when deep learning models were used. The automated segmentation of lung tumors yielded quantifiable radiomic features to train classification algorithms. Seven machine learning classifiers were compared for their accuracy in differentiating three histological subtypes of lung cancer using CT image features. The most discriminating features and most accurate classification learners were ranked.

Our study was prompted by the fact that disconnections between AI research and clinical applications exist, and we have provided a framework to close such gaps. These gaps are not due to a lack of advanced and sophisticated AI models, but to insufficient validation of the integration of existing methods using new datasets and new clinical questions. Our study validated a framework to close one such gap. Our key contributions are summarized as follows. First, we demonstrated the feasibility of directly applying a trained DL model that is publicly available to a completely new CT dataset collected for other purposes with minimal inputs from radiologists. We recognized the limitations of applying trained DL models to new datasets and proposed the incorporation radiologists’ input on approximate tumor locations for reliable and targeted segmentation. Second, we systematically examined the accuracy of this integrated approach through lung cancer subtype prediction using the segmentation results from the DL model. Third, we discerned the practical issue of unbalanced data and demonstrated that an over-sampling approach such as SMOTE (synthetic minority oversampling technique) can effectively improve the accuracy with which real clinical data are classified. Fourth, for the first time, we demonstrated that radiomic analysis is able to classify three subtypes of lung cancers with accuracy comparable to that of two-subtype classification.

We believe that our study outcomes are of more use to researchers in the applied AI and/or biomedicine communities than to those whose expertise is novel AI methodology development. This is because our objective is not to build new deep learning or machine learning approaches. Instead, we identified important, practical limitations of existing AI methods when applied to new clinical data. Clinical data need to be carefully processed to be more specific and balanced so that the performance of AI methods can be maximized.

## 2. Materials and Methods

### 2.1. Data Description

We used the previously collected and publicly available CT images in the dataset named “A Large-Scale CT and PET/CT Dataset for Lung Cancer Diagnosis (Lung-PET-CT-Dx)”, obtained from The Cancer Imaging Archive (TCIA) [15]. TCIA is an open-access information platform created to support cancer research initiatives where open access cancer-related images are made available to the scientific community (https://www.cancerimagingarchive.net/, accessed on 30 May 2021) [15]. The Lung-PET-CT-Dx dataset contains 251,135 de-identified CT and PET-CT images from lung cancer patients [16]. These data were collected by the Second Affiliated Hospital of Harbin Medical University in Harbin, Heilongjiang Province, China. Lung cancer images were acquired from patients diagnosed with one of the four major lung cancer histopathological subtypes using biopsy. Radiologist annotations on the tumor locations were also provided for each CT/PET-CT image. Each image was manually annotated with a rectangular bounding box of similar length and width to the tumor lesion using the LabelImg tool [17]. Five academic thoracic radiologists completed the annotations: the bounding box was drawn by one radiologist and then verified by the other four.

For our analysis, we only processed CT images with a resolution of 1 mm. CT scans with resolutions other than 1 mm were excluded from the analysis. We made this choice because CT images of different intervals may introduce variability in the radiomic features that complicate the interpretation of the results. A thickness of 1 mm is the most commonly acquired slice resolution in clinics [18], and such CT images were indeed the most well represented in our dataset. Therefore, focusing on 1 mm thick CT images was the most relevant choice for future clinical utilization. In some cases, a patient had more than one chest CT scan. The anatomical scan taken at the earliest time point for a given patient was included in the analysis. This earliest timepoint CT scan is referred to as the patient’s primary CT scan. We decided to exclude non-primary scans for the following reasons: non-primary scans, such as contrast-enhanced or respiratory-gated scans, do not provide radiomic features comparable to those of CT scans, and thus are not appropriate for inclusion in our analysis. Furthermore, the non-primary CT scans might have been acquired after treatment had begun, at which point potential tumor necrosis and cell-death may affect the radiomic features within the CT image. In such cases, the non-primary CT images would not truly represent the radiomic properties of the tumors, which would have changed in response to treatment. A summary of patient demographic information and tumor TNM stages is provided in Table 1 [19]. 

### 2.2. Semi-Automated Segmentation and Manual Inspection

To perform machine learning-based radiomic analysis, the Computational Environment for Radiologic Research (CERR, Memorial Sloan Kettering Cancer Center, New York, NY, USA) software platform was used to apply the trained iMRRN to automated segmentation [20]. CERR is an open-source, MATLAB-based (Mathworks Inc., Natick, MA, USA) tool with methods optimized for radiomic analysis. Using CERR, CT images in the DICOM format were converted to planC format in preparation for segmentation. Deployment of the iMRRN to the planC object enabled the segmentation of tumor ROIs and the production of a morphological mask over the tumor lesion. The Linux distribution Xubuntu 20.04 (Canonical Ltd., London, UK) was chosen for segmentation to execute the iMRRN Singularity (Sylabs, Reno, NV, USA) container.

**Figure 2:**
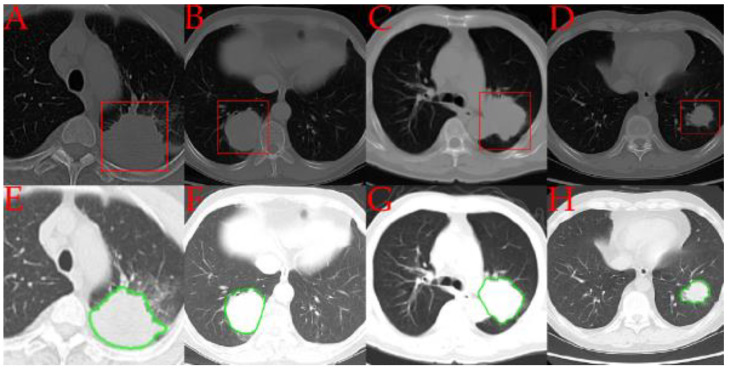
Comparisons between representative examples of tumor regions of interest annotated by radiologists (in red) (**A**–**D**) and automatically segmented by the iMRRN (in green) (**E**–**H**). Panels **A** and **E** represent a patient with adenocarcinoma (group **A**); panels **B** and **F** represent a patient with small cell carcinoma (group **B**), panels **C** and **G** represent a patient with large cell carcinoma (group **E**); panels **D** and **H** represent a patient with squamous cell carcinoma (group **C**).

A visual comparison of the iMRRN segmentations and the radiologist annotations is given in Figure 2. One image from each of the four pathologic tumor subtypes is presented. In comparison with the rectangular delineations made by the radiologist, the automated segmentations followed the contours of the tumor lesions more precisely. In these examples, the iMRRN was able to adequately segment the tumors.

**Figure 3:**
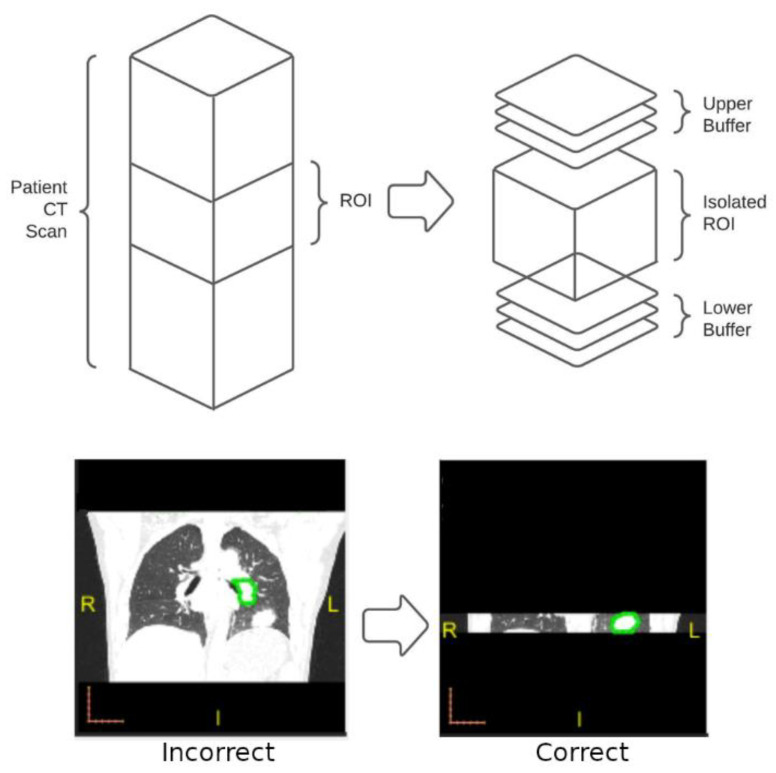
Overview of the process used to increase segmentation accuracy for images that failed initial screening. **Top**: In images for which unrestricted segmentation failed, the radiologist-defined ROI was isolated from the entire image stack (**left**) along with buffer images above and below the ROI. Automated segmentation using the iMRRN was then restricted to the target area. **Bottom**: example of incorrect segmentation of a non-tumor anatomical structure in which the iMRRN tool was used without restriction (**left**) compared with correct lesion segmentation after the field of analysis was restricted to the ROI and buffer images (**right**).

After the direct application of the iMRRN segmentation tool, the images were visually examined and compared with the manual box annotations. We found that in some patient CT scans, non-tumor structures, such as the heart, vertebrae, or sections of the patient’s couch, were mistakenly segmented as tumor nodules. Many of these structures were quite distant from where the tumors were located. To deal with such mistakes, we decided to supply the iMRRN only with CT images near the tumor locations. When performing segmentation within these focused tumor regions of interest (ROI), the iMRRN no longer erroneously segment unrelated structures. Using a Matlab program developed in house, original CT scans were trimmed by discarding the parts of the images outside the annotation boxes known not to contain the tumor lesion (Figure 3). Since there were cases in which the radiologist annotations did not cover the entire tumor, which may have led to incomplete segmentation, an upper and lower buffer were included in the ROI to increase the segmentation ROI (Figure 3, bottom panel).

### 2.3. Radiomic Feature Extraction

Using CERR methods, histogram intensity features and tumor morphology features were extracted from the segmented ROIs. These features included 17 shape features, 22 first-order features, and 80 texture features from the tumor regions in each CT scan. Texture features were further broken down into the following 5 subgroups: gray level co-occurrence matrix (26), gray level run length matrix (16), gray level size zone matrix (16), neighborhood gray tone difference matrix (5), and neighborhood gray level dependence matrix (26). Each of these features were defined mathematically in the Image Biomarker Standardization Initiative reference manual [21]. A list of extracted radiomic features is given in Supplementary Table S1. All attributes were continuous variables, and each feature was normalized to a range between zero and one so that the scales of the feature values did not affect the results. Observations containing missing values and infinite values were removed.

In order to evaluate the predictive role of 2D and 3D CT-scan features in determining tumor subtype, we initially narrowed down the analysis to the central transverse plane of the Region of Interest (ROI) for the 2D examination. Subsequently, we expanded the analysis to encompass the entire tumor mask for the 3D examination.To examine how well the center CT slice represents other slices in the same CT volume in terms of radiomic analysis, we trained several classifiers using only center slices from the CT volumes and then tested the classification accuracy on the off-center slices that were 4 mm away from the center slices. No test slices were acquired if there was no tumor lesion 4 mm away from the center.

When the 2D CT images were analyzed, only those shape features applicable in 2D (i.e., major axis, minor axis, least axis, elongation, max2dDiameterAxialPlane, and surfArea) were included. We hypothesize that these shape features are the most robust against CT scanner variation, and, therefore, will be the most important when identifying lung tumors by histological subtype.

### 2.4. Radiomic Model Building

To examine the effectiveness of supervised learning methods in classifying lung cancer subtypes using radiomic features extracted from segmented tumor CT data, we trained and tested seven classifiers. The MATLAB Classification Learner App (Statistics and Machine Learning Toolbox version 12.3) was used to perform the classification and evaluation. The training data contained the extracted radiomic features as well as the confirmed lung cancer subtypes. The following classification algorithms were considered and compared: decision tree, discriminant, naïve Bayes, support vector machine, k-nearest neighbors, ensemble, and a narrow neural network. Each of these models was trained in over fifty iterations. Five-fold cross-validation (CV) was used to evaluate the performance of each model. Five-fold CV divides the whole dataset into five subsets of equal size. Each model was trained using four subsets and then tested on the fifth subset; the process was repeated five times, and the averaged results were reported.

Three lung cancer subtypes, namely adenocarcinomas (group A), small cell carcinomas (group B), and squamous cell carcinoma (group C), were used as response variables for our analysis. Large-cell carcinomas were not included in the analysis as they were poorly represented in the dataset (only five instances). Clinically, large-cell carcinomas account for less than 10% of all lung cancer types, so omitting this particular type did not impact our study objectives.

A principal component analysis (PCA) was used to reduce data complexity [22]. A PCA works by transforming the original dataset into a new set of variables (principal components) that are linear combinations of the original features. This is a widely used technique in machine learning and is especially useful when analyzing data with many features. We used the synthetic minority over-sampling technique (SMOTE) to address the problem of class imbalance in our dataset, in which adenocarcinoma patients (*n* = 251) greatly outnumbered small cell carcinoma patients (*n* = 38) and squamous cell carcinoma patients (*n* = 61). SMOTE was used synthesized new observations using a k-nearest neighbors approach to balance the number of training observations for each histotype group [23]. The MATLAB implementation of SMOTE we used created a more balanced dataset for radiomic modeling and feature analysis [24].

Chi-square tests have been used in machine learning to select features [25]. Although chi-square tests are restricted to categorical data, discretization enables the examination of continuous variables [26]. In our study, we used chi-square tests to obtain a chi-square feature ranking. This ranking describes the degrees of association between each feature and the response variable, which is the class label for classification. Using the feature ranking, we determined which were the most important shape, texture, and first-order histogram intensity features for classifying lung cancer histological subtypes.

## 3. Results

### 3.1. Patient Demographics

We summarized the demographic and clinical information of our lung CT patient cohort (Table 1). Over-representation in the adenocarcinoma group (A) in comparison with all other histotype groups was observed. The number of large cell carcinoma observations (five) was insufficient to proceed with radiomic analysis, so these observations were not considered for the training of the classification models. Sex, age, smoking history, and TNM stage are summarized by histotype. Of note is the relatively large number of T1 observations, which denote tumor lesions less than 3 cm across.

### 3.2. Segmentation Accuracy

**Figure 4:**
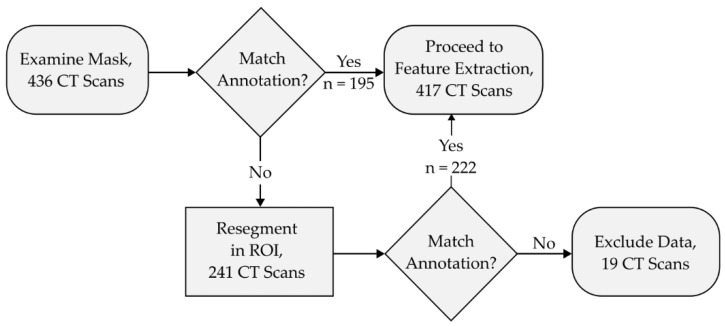
This flowchart describes the segmentation and data exclusion process. Morphological segmentation masks were manually inspected for spatial accuracy against radiologist annotations following segmentation iterations. Segmentations that matched the annotations proceeded to feature extraction. Segmentation masks that did not adhere to the radiologist annotations were segmented again within the radiologist-defined region of interest (ROI). Masks that did not match the radiologist annotations (*n* = 19) after this step are excluded from downstream analyses.

The accuracy of the automated segmentation performed by the iMRRN was first evaluated against the radiologist-defined regions of interest in 436 lung cancer images spanning various tumor subtypes and dimensions (ROI). Based on visual inspections of the morphological masks, the iMRRN initially produced accurate segmentations for 195 of the 436 (44.7%) plain CT images, and incorrectly placed the tumor region outside the radiologist’s ROI in 241 of the 436 cases (55.3%) (Figure 4). In the 241 scans that were incorrectly segmented, the iMRRN had placed the segmentation mask over a non-tumor anatomical structure outside of the radiologist-delineated ROI bounding box. This demonstrated a need for additional guidance to produce a higher number of accurate lesion contours for radiomic analysis. The 241 failed segmentations were again processed within the radiologist-defined bounds (Figure 4). An additional description of the data segmentation and exclusion process can be found in Supplementary Table S2. Of these 241 CT scans, the iMRRN segmentations matched the radiologist delineations in 222 cases. In the remaining 19 scans, the iMRRN masks did not match the radiologist’s annotations. These 19 scans, which represent different histological subtypes as well as a range of T-stages, were excluded from further radiomic analysis, resulting in an overall segmentation failure rate of 4.35%.

Overall, the restriction of the segmentation execution to the ROI produced a higher number of accurate masks compared with the unrestricted analysis. Segmentation accuracy was improved across histological subtypes and lesions of different dimensions (Table 2). We performed two different comparisons, one by histotype and one by T-stage, in which the group size was the number of patients and the successes were the number of segmented CT scans. The percentage increase in each histotype was between 25% and 130%. The segmentation accuracy for all tumor sizes was improved by different degrees in relation to the T-stage, ranging between 10% and 2100%. The earlier the T-stage was, the higher the segmentation improvement that was achieved. As expected, the greatest improvement was seen for lesions smaller than 1 cm in diameter. As is shown in Table 2, only one CT scan containing a tumor smaller than 1 cm was successfully segmented when automatic segmentation was performed on the entire CT volume. After a refined CT volume was supplied to the automatic segmentation model, 22 CT datasets containing tumors smaller than 1 cm were successfully segmented. Without this refinement, the iMRRN either incorrectly segmented a non-tumor anatomical structure or failed to produce a segmentation mask. Taken together, our data suggest that the automatic and unrestricted segmentation of relatively small tumor lesions still requires manual intervention from a trained radiologist. However, a single radiologist annotation in the coronal plane may be sufficient to guide automated software segmentation of the tumor mass even within all the corresponding transverse plane images. This form of semi-automated segmentation may represent the best of both worlds, with trained radiologists supervising precision mathematical models with high accuracy and repeatability and minimal intervention.

### 3.3. Radiomic Model Analysis Using SMOTE

From the 417 successfully segmented patient CT scans, we excluded follow-up studies to produce a training dataset of 324 unique observations. This training data included one representative segmentation mask per patient. We demonstrated how the SMOTE function rebalanced the number of observations in the training data (Table 3). Rebalancing was applied to the small cell carcinoma (B) and squamous cell carcinoma groups (C); the adenocarcinoma group (A), in contrast, was over-represented in this dataset. We applied SMOTE to the B and C groups to approximate an equal number of observations between each of the response types. Before applying SMOTE, the total number of training observations was 324, and this increased to 672 after applying SMOTE.

To assess whether machine learning classification algorithms can be used to predict histological subtypes from lung cancer CT images, we next extracted first- and second-order characteristics from the segmented images. To account for differences in the numbers of cases across histotype groups, we first examined how classification accuracy was affected by inherit variations caused by the application of SMOTE to the training data. We trained models using five separate instances of the SMOTE function and compared the resulting classification accuracy (Table 4).

### 3.4. Radiomic Analysis: Center and Center-Offset Slices

We next used our pipeline to evaluate the ability of seven machine learning models to accurately distinguish adenocarcinomas from the two other histological subtypes using 103 first- and second-order features in two dimensions using the central slide of the entire CT stack. We first conducted a two-class comparison (adenocarcinomas and squamous cell carcinomas vs. small cell carcinomas). The two-class comparison analysis represents the distinction between non-small cell carcinoma (NSCLC) and small-cell carcinoma (SCLC) cancer types. The dataset included 30 small cell carcinomas and 171 combined adenocarcinoma and squamous carcinomas. The accuracy of the classifiers in distinguishing NSCLC from SCLC ranged between 77% and 85% (Table 5). The results show minimal interference by the SMOTE function in the classification accuracy. Although the SMOTE function did not significantly improve the histological classification performance, the AUC did increase. This is possibly because the two classes of data were severely unbalanced (Table 5).

We then assessed the ability of our model system to accurately distinguish between three different tumor subtypes, namely adenocarcinomas, small cell carcinomas, and squamous cell carcinomas. As is shown in Table 3, the unbalanced three-group comparison yielded lower accuracy levels compared with the two-group comparison (ranging between 60.7% and 72.1%). However, after the groups were rebalanced using the SMOTE function, the accuracy of the ensemble, SVM, and KNN models increased to 84.3%, 85%, and 87%, respectively (Table 5).

Lastly, we applied a PCA to the 103 features to assess whether reducing the dimensionality of the variables would improve the accuracy of the classifiers. The PCA reduced the number of variables from 103 to 13 features while keeping 95% of the variability. However, the application of the PCA did not impact the performance of the algorithms when the analysis was limited to the central slide of the CT stack. The effect of the PCA on the SMOTE-resampled data is summarized in Supplementary Table S3.

### 3.5. Radiomic Feature Analysis: Whole Tumor

To account for the complex three-dimensional structures of the tumors, we next repeated the analysis using the whole stack of CT images. A total of 129 2D and 3D features were identified and tested in two- (adenocarcinomas and squamous carcinomas vs. small cell carcinomas) and three-class (adenocarcinomas vs. squamous carcinomas vs. small cell carcinomas) comparisons (Table 6). The two-class comparison analysis represents the distinction between non-small cell carcinoma (NSCLC) and small-cell carcinoma (SCLC) cancer types. In the two-class comparison, the rebalancing of the groups via the SMOTE function increased the accuracy of the models (ranges of 79.6–92.6% and 82.1–88.3% with and without the SMOTE function, respectively). As with the single image analysis, the addition of the PCA did not significantly affect the overall performance of the algorithms. In the three-class comparison, the discriminatory ability of the algorithms was slightly lower, although in this case rebalancing between the groups also increased the performance of the classifiers (unbalanced comparisons range: 67.6–89.1% versus balanced comparisons range: 73.2–92.7%). When the PCA was applied to the dataset, the SMOTE function appeared to have a greater impact than the reduction in dimensionality. For example, the SVM model had an accuracy of 76.5% and an AUC of 0.86 in the unbalanced comparison, but it has an accuracy of 92.7% and an AUC of 0.97 after the SMOTE function was applied.

### 3.6. Radiomic Feature Analysis: Three-Class Classification with Whole Tumor Features

**Figure 5:**
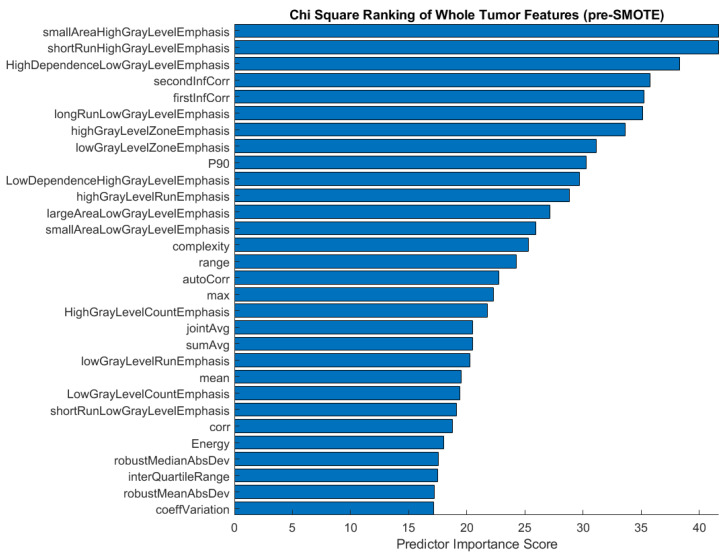
Feature ranking of the top 30 features before applying the SMOTE function to the imbalanced three-class comparison involving adenocarcinomas, small cell carcinomas, and squamous cell carcinomas.

We performed chi-square feature ranking to identify the key features in the three-class classifications using unbalanced responses. The ranking is shown in Figure 5. While texture features, particularly gray level run length matrix (GLRLM) characteristics, scored highest in importance for predicting histological subtype, shape features did not emerge as important predictors, as was previously hypothesized.

**Figure 6:**
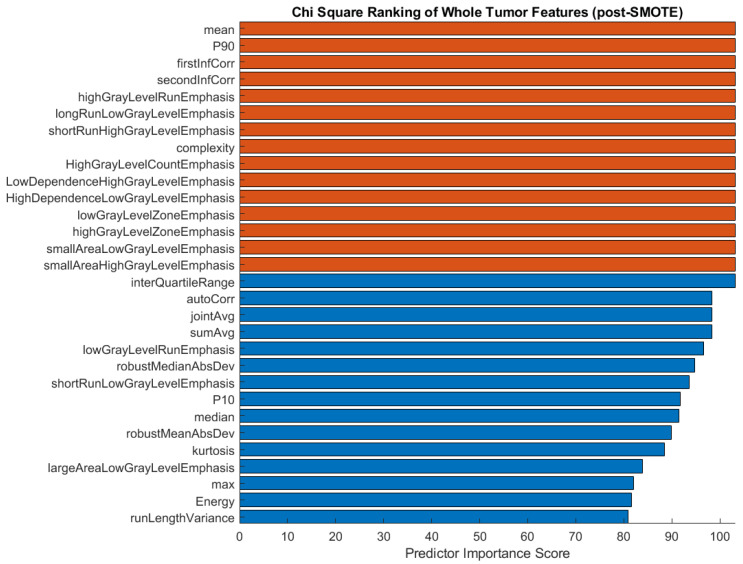
Feature ranking of the top 30 features after applying the SMOTE function to the training data in a three-group comparison. Chi-square scores with infinite values are shown in orange.

After applying the SMOTE function to the training data for three-class classification, we proceeded to analyze the chi-square feature ranking. The top 30 features were again ranked according to their predictor importance score (Figure 6). In comparison with the pre-SMOTE feature ranking (Figure 5), the post-SMOTE feature ranking places a higher emphasis on texture features, particularly GLRLM and GLCM features, in addition to first-order histogram intensity features. These features have infinite value predictor importance scores, indicating the strongest relationship between these characteristics and histological subtype classification.

## 4. Discussion

Radiomic analyses provide valuable quantitative descriptions of medical images and have great potential to be used clinically for the improved management of cancer patients [27]. One bottleneck obstructing the clinical application of radiomics in cancer diagnosis and treatment is the need for manual tracing of the tumor by a certified radiologist. In this study, we showed that a pre-trained deep learning segmentation model with minimal input from radiologists on tumor locations can be used to replace the tedious manual segmentation of lung tumors.

We examined three primary types of lung cancer in our analysis. Lung adenocarcinoma, lung squamous cell carcinoma, and small cell lung cancer exhibit distinct physical characteristics. Adenocarcinoma is the most common type and appears as irregular glands or clusters of cells, resembling glandular tissue. It typically develops in the outer regions of the lungs and is more common in non-smokers and in women. In contrast, squamous cell carcinoma is characterized by cancerous cells resembling flat, thin squamous cells arranged in layers. It commonly arises in the central airways, such as the bronchi, and is strongly associated with smoking, particularly in male smokers. Small cell lung cancer is characterized by small, round cancer cells with minimal cytoplasm that grow in clusters [28]. By carefully analyzing the CT images, radiologists can identify specific patterns associated with each type of lung cancer.

To our knowledge, our study is the first that has attempted to classify three histological subtypes of lung cancer using clinical CT/PET images. Li et al. made an attempt to classify the same three subtypes; however, their analyses were primarily binary in nature. This is because their results focused solely on comparing classification accuracies between two out of the three subtypes, without testing the accuracy of distinguishing all three subtypes from each other [29]. Every other study has classified only two subtypes (either adenocarcinoma versus squamous cell carcinoma [30,31,32] or small cell lung cancer versus non-small cell lung cancer). Our best performing model was the support vector machine, which achieved a classification accuracy of 92.7% with an AUC of 0.97 when the three lung cancers subtypes were distinguished. The SVM and ensemble models performed the best when two classes (small cell lung cancer versus non-small cell lung cancer) were considered, both achieving an accuracy of 92.6% with an AUC of 0.98. Our models outperformed those used in previous studies [32].

Our analysis provides important insights into how the proposed framework can be contextualized and used for radiomic analysis. First, although automated segmentation algorithms such as the iMRRN are designed to operate without prior information concerning the location of the tumor lesion, we found that segmentation accuracy was improved when the general location of the tumor was provided (Table 3). The annotation can be as simple as the index of the slice which contains the tumor. A deep learning (DL) model can then be applied to the tumor-adjacent slices to remove the need to search through the entire stack for the tumor. The latter method was shown by our data to have a higher rate of misidentification of the tumor lesion. As this process will only require labeling a single tumor slice, this approach requires very limited effort from the radiologist. Thus, it may boost the use of automated DL segmentation and radiomics in oncology. From a clinical perspective, developing radiomics-based tools that can predict tumor histology may spare patients from invasive procedures and help physicians capture histological changes that may emerge in response to targeted treatments [33,34].

A second important issue that emerged from our analysis is the role of an unbalanced dataset, which truly poses challenges in radiomic analysis. When working with retrospective clinical samples, it is common for a dataset to contain unequal numbers of subjects across comparison groups. However, it is also known that many machine learning methods are sensitive to unbalanced data, as the minority classes may not be learned as sufficiently as the dominant classes. One should carefully examine the distribution of data before applying machine learning based radiomic analysis because unsatisfying results could partially stem from the underrepresentation of some classes. This is especially true for multi-class classifications, in which samples can be significantly skewed. As Table 6 shows, over-sampling increased the accuracy of the two-class classification by a few percentage points (up to 4%), while more significant improvements in the accuracy of the models were seen in the three-class classification (up to 16.2%). These results are comparable to other lung radiomic studies that have demonstrated increased classification performance after applying re-sampling techniques [35].

Lastly, effective classification methods rely on features that are informative and discriminating across compared groups. Radiomic features are divided into distinct classes: shape features, sphericity and compactness features, histogram-based features, and first- and second-order features. Shape features include geometric and spatial characteristics such as size, sphericity, and the compactness of the tumor. Sphericity and compactness are features known to have strong tumor classification reliability [36]. First-order characteristics are features that describe pixel intensity values and may be expressed as histogram values. Histogram-based features have been shown to have a high degree of reliability in radiomic studies [36]. Second-order features, or texture features, rely on statistical relationships between patterns of gray levels in the image. The gray level run length matrix and gray level zone length matrix features each describe homogeneity between pixels and have been shown to be reliable second-order features [37]. Our study showed that 3D CT data outperform single 2D CT data by up to 5% when their radiomic features are used in classification. There are several factors that might cause this. First, 3D CT data provides a richer set of radiomic features, such as the true shape and size information of the three spatial dimensions described above. Second, tumor morphometric and texture characteristics are subject to spatial heterogeneity, which can only be captured by 3D features. Two-dimensional texture features may not be sufficient to accurately describe spatial heterogeneity. However, if clinical 3D CT data are unavailable, 2D radiomic analysis can still be used to achieve useful classification with decent accuracy.

Improving the accuracy of such classifications will rely on the selection of discriminating features. This study utilized each of the shape, first-order, and texture features available in CERR, as these are shown to be robust against differences in image acquisition techniques [38]. Incorporating clinical features such as age, sex, weight, and smoking history are likely to improve classification accuracy as these have been shown to correlate with risk for lung cancer [39].

## 5. Conclusions

This study demonstrated a successful application of the deep learning method iMRRN to the segmenting of independent lung CT data and identified a procedure to make automatic segmentation more accurate. The direct use of segmentation with existing deep learning models leads to classification accuracy comparable with that typically achieved in published studies. The necessity of balancing data samples was demonstrated, as was a suitable data balancing method. The feasibility of performing classifications with three classes was shown by systematically comparing various machine learning methods. Overall, we demonstrated that the classification of subtypes of lung cancer can be semi-automated with minimal radiologist intervention in terms of segmentation and radiomic analysis.

## Figures and Tables

**Table 1:** Summary of the demographic and clinical information of the 355 patients with CT images in the TCIA dataset.

Subgroup	A—Adenocarcinoma	B—Small Cell Carcinoma	E—Large Cell Carcinoma	C—Squamous Cell Carcinoma	*p*-Value
Sex					<0.01
M	118	21	4	47	
F	133	17	1	14	
Age					0.604
Median	62	63.5	63	61	
Range	28–63	32–77	41–72	47–90	
Smoking History					<0.01
S	91	18	3	43	
NS	160	28	2	18	
T-Stage					<0.01
T1	139	10	1	19	
T2	74	13	0	19	
T3	29	11	1	16	
T4	9	4	3	7	
N-Stage					<0.01
N0	145	5	1	33	
N1	58	12	1	14	
N2	5	0	1	2	
N3	43	21	2	12	
M-Stage					<0.01
M0	161	25	0	44	
M1	90	13	5	17	

M = male; F = female; S = smoking; NS = non-smoking.

**Table 2:** Summary of segmentation results by histological group and by T-stage before and after the restriction of the image to the spatial range based on the annotation bounding box.

Histotype Symbol	Group Size	Group Description	Pre-Restriction Successes	Post-Restriction Successes	Percentage Increase
A	251	Adenocarcinoma	127	292	130%
B	38	Small cell carcinoma	27	47	74%
E	5	Large cell carcinoma	4	5	25%
C	61	Squamous cell carcinoma	37	73	97%
**T-Stage**	**Group Size**	**Group Description**	**Pre-Restriction Successes**	**Post-Restriction Successes**	**Percentage Increase**
1a	12	Tumor smaller than 1 cm	1	22	2100%
1b	29	Tumor smaller than 2 cm	8	33	312%
1c	128	Tumor smaller than 3 cm	52	158	203%
2	106	Tumor smaller than 5 cm	69	116	68%
3	57	Tumor smaller than 7 cm	46	67	45%
4	23	Tumor larger than 7 cm	19	21	10%

**Table 3:** Summary of the number of training observations by histotype class both before and after applying SMOTE.

	A	B	C	Total
Before SMOTE	226	38	60	324
After SMOTE	226	224	222	672

**Table 4:** Summary of the classification results for five instances of models trained with SMOTE-resampled observations.

Model	Iteration 1	Iteration 2	Iteration 3	Iteration 4	Iteration 5
Tree	80.10%	80.10%	78.30%	79.60%	79.30%
Discriminant	73.20%	72.90%	74.40%	73.10%	70.40%
Naïve Bayes	71.40%	70.80%	73.10%	69.90%	71.30%
SVM	92.70%	93.20%	91.70%	92.40%	87.80%
KNN	89.00%	90.30%	89.90%	89.90%	89.60%
Ensemble	89.00%	90.60%	90.30%	89.90%	88.10%
Narrow Neural Network	83.00%	83.80%	83.00%	84.20%	83.80%

SVM = support vector machine; KNN = K-nearest neighbors.

**Table 5:** Summary of the classification results using features extracted from the central axial slice of the tumor volume from the patients’ primary CT scans before and after SMOTE resampling.

	Two-Class Classification			
Classification Model	Pre-SMOTE CV Accuracy	Post-SMOTE CV Accuracy	Pre-SMOTE AUC	Post-SMOTE AUC
Tree	85.10%	74.40%	0.50	0.73
Discriminant	83.60%	77.60%	0.73	0.87
Naïve Bayes	81.60%	74.00%	0.61	0.82
SVM	85.10%	77.60%	0.50	0.84
KNN	85.10%	80.50%	0.71	0.9
Ensemble	85.10%	78.00%	0.50	0.86
Narrow Neural Network	77.1%	78.30%	0.48	0.80
	**Three-Class Classification**			
**Classification Model**	**Pre-SMOTE CV Accuracy**	**Post-SMOTE CV Accuracy**	Pre-SMOTE AUC*	Post-SMOTE AUC*
Tree	70.10%	73.30%	0.75	0.81
Discriminant	70.60%	77.50%	0.82	0.88
Naïve Bayes	68.20%	68.90%	0.81	0.85
SVM	72.10%	85.00%	0.82	0.84
KNN	71.60%	87.00%	0.84	0.97
Ensemble	71.60%	84.30%	0.77	0.93
Narrow Neural Network	60.70%	77.50%	0.65	0.78

AUC = area under curve (* adenocarcinoma is the positive class); CV = five-fold cross-validation; SVM = support vector machine; KNN = K-nearest neighbors.

**Table 6:** Summary of the classification results using features extracted from the whole tumor volume in the patients’ primary CT scans.

	Two-Class Classification			
Classification Model	Pre-SMOTE CV Accuracy	Post-SMOTE CV Accuracy	Pre-SMOTE AUC	Post-SMOTE AUC
Tree	88.30%	82.30%	0.48	0.85
Discriminant	87.30%	82.50%	0.75	0.90
Naïve Bayes	84.00%	79.60%	0.71	0.86
SVM	88.30%	92.60%	0.69	0.98
KNN	88.30%	88.70%	0.69	0.90
Ensemble	88.30%	92.60%	0.48	0.98
Narrow Neural Network	82.10%	89.90%	0.61	0.92
	**Three-Class Classification**			
**Classification Model**	**Pre-SMOTE CV Accuracy**	**Post-SMOTE CV Accuracy**	Pre-SMOTE AUC*	Post-SMOTE AUC*
Tree	76.5%	80.1%	0.84	0.87
Discriminant	73.5%	73.2%	0.86	0.87
Naïve Bayes	74.7%	71.4%	0.83	0.89
SVM	76.5%	92.7%	0.86	0.97
KNN	89.1%	89.0%	0.87	0.86
Ensemble	77.2%	89.0%	0.86	0.96
Narrow Neural Network	67.6%	83.0%	0.71	0.86

AUC = area under curve (* adenocarcinoma is the positive class); CV = five-fold cross-validation; SVM = support vector machine; KNN = K-nearest neighbors.

## Data Availability

Data are available in a publicly accessible repository. The data presented in this study are openly available in The Cancer Imaging Archive at https://doi.org/10.7937/TCIA.2020.NNC2-0461, reference number [16].

## Supplementary Materials

The following supporting information can be downloaded at: https://www.mdpi.com/article/10.3390/bioengineering10060690/s1, Table S1: Extracted Radiomic Features Using CERR; Table S2: Segmentation and Data Exclusion Process; Table S3: Classification Accuracy Before and After Applying PCA to the Training Dataset With SMOTE applied.
