# Supplementary material for: Automated Classification of Lung Cancer Subtypes Using Deep Learning and CT-Scan Based Radiomic Analysis
Source: Bioengineering (Basel). 2023 Jun 6;10(6):690. doi: 10.3390/bioengineering10060690 (PMC10295141; doi:10.3390/bioengineering10060690)
Supplement: Supplementary file 1 [file bioengineering-10-00690-s001.zip › bioengineering-2320298-supplementary.pdf]

Supplementary material

Supplementary Table S1: Extracted Radiomic Features Using CERR.

Feature Type	Number of Features	Feature Names				
Shape	17	majorAxis	minorAxis			
		leastAxis	flatness			
		elongation	max3dDiameter			
		max2dDiameterAxialPlane	max2dDiameterSagittalPlane			
		max2dDiameterCoronalPlane	surfArea			
		volume	filledVolume			
		Compactness1	Compactness 2			
		spherDisprop	sphericity			
		surfToVolRatio				
First Order	22	Min	max	mean		
		range	std	var		
		median	skewness	kurtosis		
		entropy	rms	energy		
		totalEnergy	meanAbsDev	medianAbsDev		
		P10	P90	robustMeanAbsDev		
		robustMedianAbsDev	interQuartileRange	coeffDispersion		
		coeffVariation				
GLCM	26	Energy	jointEntropy	jointMax	jointAvg	
		jointVar	contrast	invDiffMom	invDiffMomNorm	
		invDiff	invDiffNorm	invar	dissimilarity	
		diffEntropy	diffVar	diffAvg	sumAvg	
		sumVar	sumEntropy	corr	clustTendency	
		clustShade	clustPromin	haralickCorr	autoCorr	
		firstInfCorr	secondInfCorr			
GLRLM	16	grayLevelNonUniformity	grayLevelNonUniformityNorm			
		grayLevelVariance	highGrayLevelRunEmphasis			
		longRunHighGrayLevelEmphasis	longRunEmphasis			
		longRunLowGrayLevelEmphasis	lowGrayLevelRunEmphasis			
		runEntropy	runLengthNonUniformity			
		runLengthNonUniformityNorm	runLengthVariance			
		runPercentage	shortRunEmphasis			
		shortRunHighGrayLevelEmphasi s	shortRunLowGrayLevelEmphasi s			
GLSZM	16	smallAreaEmphasis	largeAreaEmphasis			
		grayLevelNonUniformity	grayLevelNonUniformityNorm			
		sizeZoneNonUniformity	sizeZoneNonUniformityNorm			
		zonePercentage	lowGrayLevelZoneEmphasis			
		highGrayLevelZoneEmphasis	smallAreaLowGrayLevelEmphasi s			
		smallAreaHighGrayLevelEmphasi s	largeAreaLowGrayLevelEmphasi s			
		lageAreaHighGrayLevelEmphasis	grayLevelVariance			
		sizeZoneVariance	zoneEntropy			
NGTDM	5	Coarseness	contrast	busyness	complexity	strength
NGLDM	26	LowDependenceEmphasis				
		HighDependenceEmphasis				
		LowGrayLevelCountEmphasis				
		HighGrayLevelCountEmphasis				

		LowDependenceLowGrayLevelEmphasis
		LowDependenceHighGrayLevelEmphasis
		HighDependenceLowGrayLevelEmphasis
		HighDependenceHighGrayLevelEmphasis
		GrayLevelNonuniformity
		GrayLevelNonuniformityNorm
		DependenceCountNonuniformity
		DependenceCountNonuniformtyNorm
		DependenceCountPercentage
		GrayLevelVariance
		DependenceCountVariance
		Entropy
		Energy

GLCM = gray level co-occurrence matrix; GLRLM = gray level run length matrix; GLSZM = gray level size-zone matrix; NGTDM = neighborhood gray tone difference matrix; NGLDM = neighborhood gray level dependence matrix

Supplementary Table S2: Segmentation and Data Exclusion Process

Step	Number of Scans
Examine Mask	436
Match with Annotation	195
Resegmentation in ROI	241
Match with Annotation	222
Included Scans	417
Excluded Scans	19

Supplementary Table S3: Classification Accuracy Before and After Applying PCA to the Training Dataset With SMOTE Applied.

		**2-Class Classification**		
**Classification Model**	**Pre-PCA CV Accuracy**	**Post-PCA CV Accuracy**	**Pre- PCA AUC**	**Post- PCA AUC**
Tree	84.50%	84.50%	0.85	0.83
Discriminant	**	82.50%	**	0.88
Naïve Bayes	81.60%	82.90%	0.85	0.88
SVM	85.40%	82.90%	0.88	0.91
KNN	86.70%	86.30%	0.94	0.93
Ensemble	87.80%	87.60%	0.94	0.93
Narrow Neural Network	83.60%	77.80%	0.83	0.79
		**3-Class Classification**		
**Classification Model**	**Pre- PCA CV Accuracy**	**Post- PCA CV Accuracy**	**Pre-PCA AUC***	**Post- PCA AUC***
Tree	80.20%	80.10%	0.87	0.87
Discriminant	60.90%	73.20%	0.85	0.86
Naïve Bayes	65.50%	71.40%	0.86	0.89
SVM	90.30%	92.70%	0.9	0.97
KNN	89.60%	89.00%	0.88	0.86
Ensemble	90.80%	89.00%	0.98	0.96
Narrow Neural Network	87.90%	83.00%	0.91	0.86

AUC = area under curve, *Adenocarcinoma as the positive class, **CV accuracy not available
CV = 5-Fold Cross-Validation; SVM = Support Vector Machine; KNN = K-Nearest Neighbors
